# The Superficial Musculoaponeurotic System of the Face: A Model Explored

**DOI:** 10.1155/2013/794682

**Published:** 2013-11-04

**Authors:** M. Broughton, G. M. Fyfe

**Affiliations:** Faculty of Health Sciences, Curtin University, P.O. Box U1987, Perth, WA 6845, Australia

## Abstract

Regional differences in the integument of the body are explained, at least in part, by differences in fascial arrangements. In the face, where the skin is more mobile due to the action of the underlying facial muscles, fascial organisation is important for support and separation of muscle groups. This study used bequeathed cadaver material to investigate a current model of the SMAS proposed by Macchi et al., the original boundaries of which were explored and extended using both histology and gross dissection. As a clearly identifiable structure spanning the lateral and midface, the SMAS in the specimen supported the model proposed by Macchi et al. The three main findings that support the model were the layered morphological appearance of the SMAS, its progression from fibrous to aponeurotic in a lateral to medial direction, and the enveloping of the zygomaticus musculature. Extension beyond the proposed model into the temporal region was observed, but nasal and forehead regions showed no evidence of SMAS, while its presence in the cervical platysma region remained inconclusive. Fascial and soft tissue variability was considerable within facial regions of the examined specimen, helping to explain the debate around the SMAS in the literature.

## 1. Introduction

In 1976, a superficial musculoaponeurotic system (SMAS) was described in the parotid and cheek regions of the face, dividing superficial and deep adipose tissue [1]. Since then, the definitions and descriptions of the SMAS have been the subject of much debate in the literature. Comprehensive knowledge of regional variation within the face is important for the application of surgical facelift techniques [2, 3]. However, terminology, definitions, and descriptions of SMAS morphology are inconsistent [4–7], with some studies even questioning its existence [8, 9]. 

Histological studies have failed to reach consensus regarding investiture of the zygomatic musculature by the SMAS [2, 4]. Macchi et al. [2] argued that the SMAS invested the zygomaticus muscle group, while Gassner et al. [4] disagreed. The existence of the SMAS separate from the parotid fascia was more readily agreed upon [7, 10, 11], although earlier studies remained inconclusive [9, 12].

Fascial relationships of the platysma muscle are also inconclusive. Earlier studies which identified both superficial and deep fascial layers of the muscle [6] have been supported [8]; although investigative methods varied, the superficial fascial layer has not always been identified as a separate layer [13].

The concept of an SMAS is generally accepted in aesthetic surgery and applied in techniques to correct ptosis of facial fat in areas prone to aging, whereby the SMAS is drawn up and fixed to lift more superficial muscular and dermal structures [14]. Two decades ago, it was reported that at least fifty percent of face-lift procedures included some sort of SMAS dissection [7], highlighting the importance of clarifying the morphology and spatial relationships of the fascial layers of the face and particularly the SMAS. Various SMAS techniques related to aesthetic surgery continue to be described in the literature [14–16].

Macchi and colleagues were the first to present the SMAS as a continuous layer extending from the parotid region to the nasolabial fold with progressive regional thinning. This study used histological and gross dissection techniques to investigate whether or not the SMAS model proposed by Macchi et al. [2] was supportable and if it could be extended to include other facial regions. Please see Macchi et al. [2] for a detailed description of the model.

## 2. Materials and Methods

Full skin thickness samples used in the study were from an 85-year-old male perfused cadaver obtained by Curtin University via a local donor bequest programme. The cadaver was perfused with a 5% formalin solution. Because of greater lividity and skin folding on the right-hand side of the neck, the left side of the face was used for all histological samples and subsequent dissection.

### 2.1. Tissue Preparation and Histological Examination

**Figure 1:**
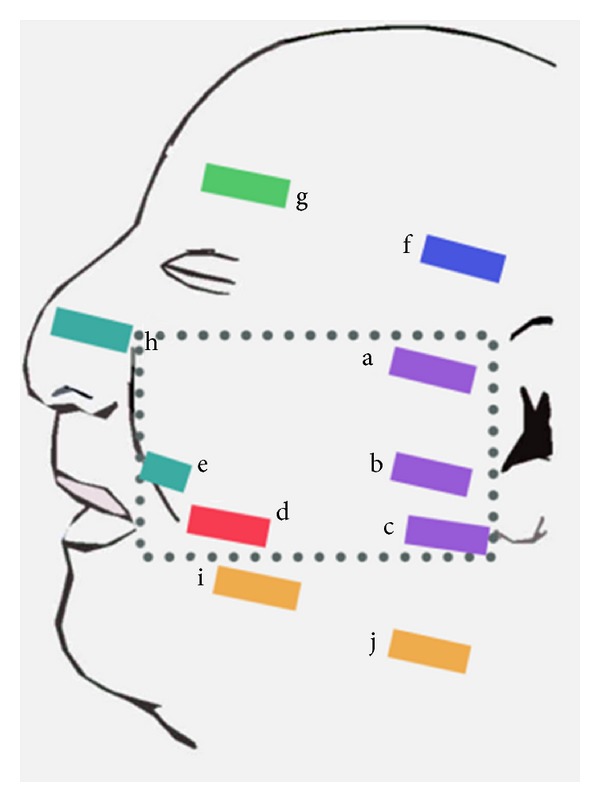
Location of tissue samples on specimen, with reference to orbitomeatal plane. Dotted region corresponds to Macchi's boundaries. a—zygomatic, b—superior parotid, c—inferolateral parotid, d—buccal, e—nasolabial fold, f—temporal, g—forehead, h—nasal, i—facial platysma, and j—cervical platysma.

Ten facial regions to be excised for histological examination were determined from the underlying bony landmarks and macroscopic features of the skin surface. Five regional samples corresponded to the model proposed by Macchi et al. [2]: zygomatic, parotid (superior and inferolateral), buccal, and nasolabial fold. Five further samples extended beyond the model: temporal, forehead, nasal, and platysma (facial and cervical) (Figure 1). Two adjacent 15 mm × 3 mm × 15 mm (length, width, and depth) blocks of tissue were excised from each region.

Excised tissue was processed, embedded in paraffin wax, sectioned at 5 *μ*m, and stained with hematoxylin and eosin (H&E) and Masson's Trichrome. Images of histological slides were taken at 40x magnification with a ProgRes C14 camera attached to a light microscope. Regional images were merged using Adobe Photoshop CS5. The resulting scaled micrographs were interpreted visually to compare them with Macchi's model, identify SMAS, and measure the depth of the SMAS from the skin surface. Measurements of structures of interest were made, converted to the scale of the image, and averaged to give a final result. All measurements correspond to the average thickness of the soft tissue structure (mm) ± standard error of the mean.

### 2.2. Gross Dissection

Dissection of the lateral aspect of the face was performed using the boundaries outlined by Macchi et al. [2]. Skin was removed from the bordered area, and subcutaneous fat and fascia were removed in successive thin layers where possible. Zygomaticus muscle group and the deep inferior region of the orbicularis oculi muscle were reflected. Dissection was continued inferiorly across the lower facial and cervical platysma and superiorly to include the temporal sample. Digital images were taken throughout to record the results of the dissection.

## 3. Results

### 3.1. Macchi's Model

**Figure 2:**
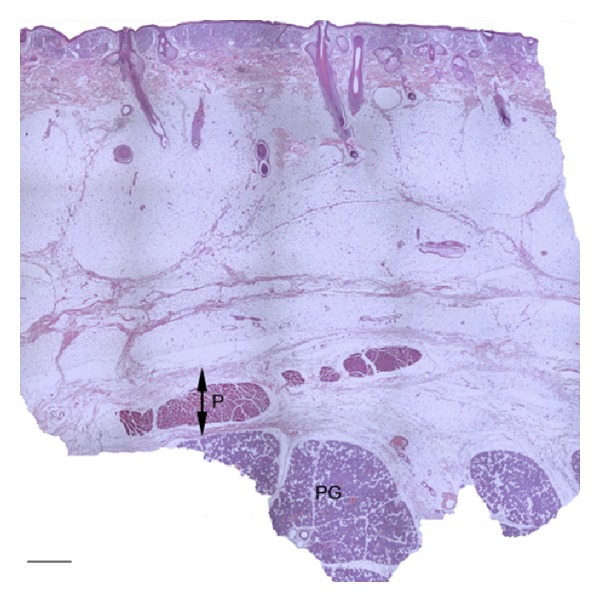
Superior parotid histological section. Macroscopic view of the parotid section in its entirety. P—platysma muscle, PG—parotid gland (H&E), and double-ended arrow—SMAS. Scale bar 1 mm.

Histologically, connective tissue layers consistent with Macchi's descriptions of the SMAS were seen in both the parotid (Figure 2) and zygomatic tissue samples. Gross dissection of the buccal region also revealed the SMAS, although the nasolabial fold region showed no evidence of SMAS when explored by either method. Region specific characteristics are described below.

### 3.2. Parotid

**Figure 3:**
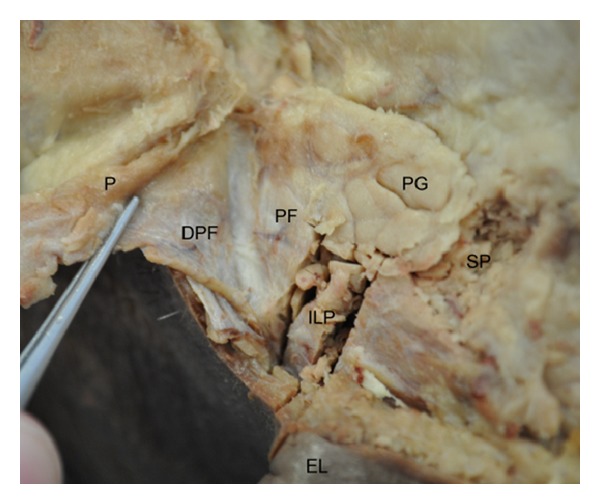
Inferolateral aspect of the face, separation of platysma muscle and parotid fascia. PG—parotid gland, PF—parotid fascia, DPF—deep platysma fascia, P—platysma, ILP—inferolateral parotid section, SP—superior parotid section, and EL—ear lobe for orientation.

In the parotid samples, the SMAS was superficial to two distinct fibrous layers, the deep platysma fascia and the parotid fascia. The platysma muscle and the SMAS in the parotid region could be raised as a continuous sheet and could be easily separated from the parotid fascia (Figure 3). The SMAS was thinner in the superior parotid sample (0.419 ± 0.065) compared with the inferolateral (0.455 ± 0.097) (Table 1). All measurements correspond to the average thickness of the soft tissue structure (mm) ± standard error of the mean. The platysma extended high into the face of the examined specimen, as evident in the inferior parotid tissue sample.

### 3.3. Zygomatic

The superficial aspect of the zygomatic musculature was enveloped by the SMAS in the midface, which extended beyond the medial border of the zygomaticus musculature, superficial to branches of the facial nerve. 

### 3.4. Buccal and Nasolabial Fold

The buccal tissue sample contained long fibrous septae, which gave the region a distinctly polygonal appearance histologically. In contrast, the nasolabial fold had minimal fibrous septae on the lateral aspect. Although small bundles of muscular fibers were present in the superficial dermal tissue of the nasolabial fold region, the majority of dense muscle fibers were deep. However, only the buccal region showed evidence of the SMAS histologically.

### 3.5. Beyond the Model

The SMAS was extended into the temporal region and was also present in the facial platysma tissue sample. The SMAS was not present in the forehead or nasal regions of the examined specimen. Forehead samples were characterised by obliquely oriented fibrous septae traversing subcutaneous fat. The frontalis muscle had little superficial muscular fascia but did have some intramuscular fascia, similar to that seen in the temporalis muscle. Also similar to the temporal region was the close adherence of the superficial fascial tissue to the muscle surface of frontalis. Nasal samples had minimal subcutaneous adipose tissue separating the layers. Region-specific characteristics of the temporal and cervical platysma regions are described below.

### 3.6. Temporal

Average epidermis and dermis thickness for the temporal and zygomatic regions were 1.589 ± 0.122 and 1.569 ± 0.047, respectively. Therefore, both temporal and zygomatic tissue samples had similar epidermal to dermal thickness. 

### 3.7. Platysma

An irregular array of fibrous septae was present in the superficial adipose layer of the facial and cervical platysma tissue samples. The platysma muscle had fascia both superficial and deep to its surface. The most inferior facial platysma sample was clearly continuous with the SMAS within the boundaries of Macchi's model, as was also seen in the parotid tissue sample, but, in the cervical platysma sample, the SMAS and its spatial relationships were difficult to discern due to a thick layer of subcutaneous adipose tissue present in the cadaver specimen. 

### 3.8. Summary of Findings

The SMAS was evident in the parotid, zygomatic, buccal, temporal, and facial platysma regions. Forehead, nasal, and nasolabial fold regions of the face showed no evidence of SMAS. The zygomaticus musculature of the midface was enveloped by the SMAS, which had a layered appearance histologically and became progressively more aponeurotic and hence thinner towards the medial aspect of the face. 

Fibrous septae in the superficial adipose layer of the buccal and forehead tissue samples were organised in appearance; obliquely oriented septae were present in the forehead, whereas, in the buccal region, septae were long and vertical in orientation. The parotid and platysma samples displayed no particular organisation of fibrous septae. 

## 4. Discussion

The SMAS clearly enveloped the zygomaticus musculature in the examined specimen in support of the enveloping terminology used in the model proposed by Macchi et al. [2] to describe the relationship of the SMAS with the zygomatic musculature. Similarly, facial nerve branches medial to zygomaticus in the examined specimen were deep to the SMAS layer, although the temporal branch was not found in our specimen during dissection. Macchi et al. [2] considered the SMAS to be the facial extent of the superficial temporal fascia. In our study, the interpretation of histological evidence from our samples, including the similar thickness and morphology of the SMAS across zygomatic and temporal samples, supports this view.

Similar to Macchi et al. [2], and as others have also reported [7, 10, 11], the SMAS and parotid fascia were identified as two separate entities in our specimen. Macchi and colleagues [2], however, reported minimal evidence of the platysma muscle in parotid tissue samples examined, while, in our study, the platysma muscle was extensive in the examined specimen. Extent of the platysma muscle into the face is variable [17], therefore, the extent of the muscle in our specimen was not outside normal range, although more extensive than those used in Macchi's study, possibly due to differences in the method and the use of select cadavers [18]. Absence of platysma will result in thinner appearance of SMAS. Macchi et al. [2] reported an average SMAS thickness of 0.386 ± 0.113 mm; we attained a measurement of 0.455 ± 0.097 mm. From a larger sample of eight cadavers, Macchi's figure is representative of a wider age range. In addition, a recent study by Erian and Shiffman [19] further highlighted the significant individual variation of the lower region of SMAS, which is supported by our results.

### 4.1. Alternative Interpretations of Facial Morphology

In our study, the facial musculature in the nasolabial fold region was much deeper than that of any other facial regions, and our results found no evidence of the SMAS, supporting Pessa and Brown's [20] assertion of poor connection between the deeper musculature of the mouth and the SMAS. Substantial amounts of subcutaneous adipose tissue in our specimen would change the relative depths of the underlying tissue layers [21]. Erian and Shiffman [19] included subcutaneous adipose tissue in their three-part model of the SMAS and proposed the terminological change “SMA-Fatty-S”, comprised of a fibroaponeurotic part, the superficial adipose layer, and facial musculature. Our results support this proposed refinement of the current view of the SMAS.

Human variability and the effects of aging both contribute to the appearance, spatial relationships, and nature of the face and its underlying soft tissue structures [3, 22]. Mendelson et al. [23] showed that distension of the facial ligaments connecting the underlying masseteric fascia to the overlying platysma muscle leads to the stretched appearance of the facial platysma muscle in older people, which consequently adds to the macroscopic visibility of the nasolabial fold as we age. Though the SMAS itself was not present in the nasolabial region of the specimen examined, Mendelson et al. [23] highlight the close relationship of nearby structures of the face and that the movement of one region can have impact on another region nearby, suggesting that the SMAS does not have to be present to have an effect on the macroscopic appearance of the nasolabial fold.

Our results support the findings of Raskin and LaTrenta [21] who described short, dense fibrous septae in forehead and temporal regions and long, loose septae in the neck and cheek regions. The loose arrangement of fibrous septae and a higher amount of subcutaneous fat tissue in areas such as the cheek and neck as evident in our specimen would provide less support in these regions in comparison to the forehead, where fibrous septae are distinct [21]. Furthermore, Besins [3] considered the midface and neck to be moving regions, whereas the nasal and forehead regions were fixed. Together, this would explain why both the cheek and neck show characteristic signs of aging such as ptosis of the malar superficial fat tissue and banding of the platysma [22]. The distinct subcutaneous tissue arrangement observed in the nasal and forehead regions may also be due in part to their development, as both regions develop from the same singular facial primordia [24]. Further investigations into fetal development of the fascial planes of the face would provide insight into the underlying support structures present in the adult face and the changes that occur with age.

### 4.2. Limitations

Our study was limited by the use of only one specimen for examination and the superficial tissue depth in some regions. Further, we were unable to investigate the relationship of the deeper aspects of some of the facial musculature such as the zygomaticus muscles with underlying structures beyond the boundaries of the model within the scope of the project. The restricted area and size of histological samples leave room for additional histological examination, whereby deeper tissue sections would aid in a better overall interpretation of the facial soft tissue. 

## 5. Conclusion

As a clearly identifiable structure spanning the lateral and midface, the appearance of the SMAS in this study supported the model proposed by Macchi et al. [2] in three main ways: (1) the layered morphological appearance of the SMAS, (2) its progressively fibrous to aponeurotic nature, and (3) enveloping of the zygomaticus musculature. Beyond the boundaries of Macchi's study, the SMAS was seen in temporal but not nasal or forehead regions. The presence of the SMAS in the cervical platysma region was inconclusive, impeded by a deep subcutaneous adipose layer.

Variability within the platysma muscle influences the extent and thickness of the SMAS. In our specimen, the platysma traversed high into the face, with a significant amount of muscular fibers evident in the most lateral aspect which influenced the appearance and thickness of the SMAS in the lateral aspect of the face.

Closely related facial regions exhibit morphological differences in fascial, muscular, and adipose tissues; variability in facial musculature is well known although related fascia variance is poorly documented, which could explain the contradictions in the literature. By increasing sample size and extending the coverage of tissue sample regions to include two perpendicular planes of reference containing a cross sectional area, the nature of fascial variation within the face and the implications of such variance would be extended further.

## Figures and Tables

**Table 1:** Measured thickness of SMAS in parotid region.

Tissue sample	Mean thickness of SMAS layer (mm)
Inferolateral parotid	0.455 ± 0.097
Superior parotid	0.419 ± 0.065
